# A Case Report of MuSK Antibody-Positive Myasthenia Gravis

**DOI:** 10.7759/cureus.61820

**Published:** 2024-06-06

**Authors:** Dinusha Gayathri, Shanika Nandasiri, Gamini Pathirana

**Affiliations:** 1 General Medicine, Teaching Hospital Peradeniya, Peradeniya, LKA; 2 Neurology, Colombo North Teaching Hospital, Colombo, LKA; 3 Neurology, National Hospital Sri Lanaka, Colombo, LKA

**Keywords:** rare subtype, seronegative, rituximab, musk antibody positive, myasthenia gravis

## Abstract

Myasthenia gravis (MG) is characterized by muscle weakness and fatigability. The presence of autoantibodies against the acetylcholine receptors (AChR) at the neuromuscular junction, which impairs neuromuscular transmission, is the hallmark of the disease. However, a minority of patients have antibodies against muscle-specific tyrosine kinase (MuSK), which is referred to as MuSK myasthenia gravis (MuSK-MG).

We present the case of a 56-year-old female patient presenting with progressive dysphagia, slurred speech, and fatigable ptosis. She had a positive icepack test and a positive repetitive nerve stimulation test (RNST). Her AchR antibodies were negative, and the MuSK antibodies were positive. Her clinical response to pyridostigmine was unsatisfactory, but she had a good recovery with rituximab.

Even though MuSK-MG is rare, it is an important diagnostic consideration, particularly in patients presenting with atypical symptoms or lacking AChR antibodies and in patients who have a poor response to conventional treatment. Acetylcholinesterase inhibitors, corticosteroids, immunosuppressants, and newer biologic agents targeting B cells are some of the treatments.

## Introduction

Muscle-specific tyrosine kinase (MuSK) antibody-positive myasthenia gravis (MG) is a rare but more severe subtype of MG that occurs acutely and affects mainly the bulbar and facial muscles. However, ocular manifestations, including horizontal gaze palsy, have been demonstrated in the early stages of the disease [1]. It has rapid progression, and early respiratory failure is frequent. Muscle atrophy is an unusual but distinct feature of MuSK antibody-positive myasthenia gravis (MuSK-MG), primarily affecting facial muscles and the tongue. It can also occur in the shoulder girdle, limb, and paraspinal muscles and may be reversible with treatment [1-3].

Its prevalence varies among countries and ethnic groups, with a higher percentage in Europe, and is predominantly seen in females [1,4]. Most often, it presents before the age of 40 years. Seronegative MG is a term used to describe generalized MG where no acetylcholine receptor (AChR) antibodies are detectable. About 5% to 40% of patients with seronegative generalized MG are diagnosed with MuSK-MG [1,5,6]. There is no prevalence data of MuSK-MG available from South Asia except for one article from Sri Lanka [6]. Per this article, MuSK-MG is seen in about a fifth of generalized seronegative MG patients in Sri Lanka with clinical features consistent with those described in Caucasians.

The use of acetylcholinesterase inhibitors for symptomatic treatment is generally unsatisfactory in MuSK-MG patients. Steroids and immunosuppression therapy have a better response, and monoclonal antibodies such as rituximab therapy showed greater and sustained outcomes in MuSK-MG patients [1,7,8].

## Case presentation

**Figure 1:**
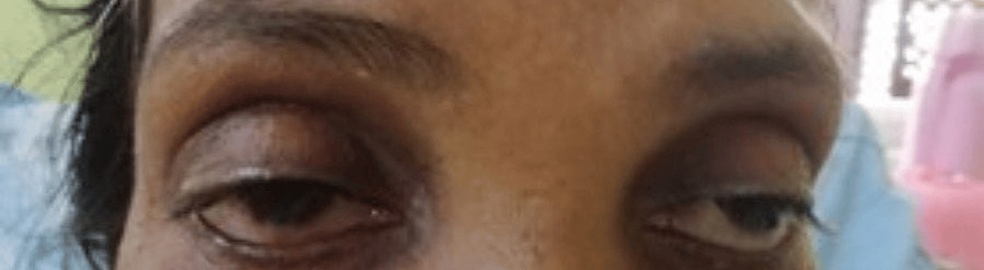
Before the ice pack test

**Figure 2:**
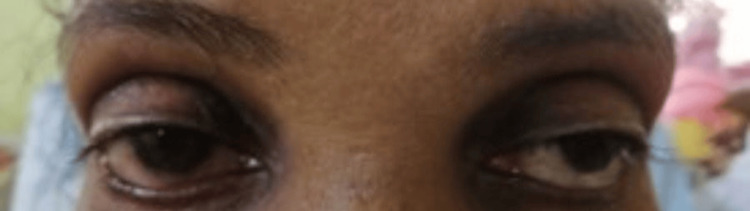
Improvement after the ice pack test

A 56-year-old previously healthy patient developed dysphagia and slurring of speech, which gradually progressed over four months. Initially, she had dysphagia for solids, and later it progressed to liquids, and ultimately, she had difficulty swallowing her saliva. She found it difficult to initiate swallowing and had food regurgitated from the nose. She also noted a change in her voice with slurring of speech. In addition, she had difficulty raising her arms to comb her hair. However, there was no lower limb weakness or double vision. At first, the symptoms were worsening towards the evening, and later, symptoms persisted throughout the day. Examination revealed bilateral (B/L) partial ptosis with a positive ice pack test (Figures 1-2).

Bilateral pupils were equally reactive and had right-sided abduction failure. Her speech had a nasal quality, and B/L palatal movements were reduced with weakness in B/L orbicularis oculi, buccinators, orbicularis oris, and platysma. She had weak neck extensors with reduced proximal muscle power (4/5) in B/L upper limbs with reduced reflexes. The lower limbs were normal and had no cerebellar signs. The tongue examination was normal and did not show evidence of atrophy. Other system examinations were normal. Before admission, she had undergone an upper gastrointestinal endoscopy (UGIE) for dysphagia, which revealed mild antral gastritis, and an ear, nose, and throat evaluation found reduced movements of the left side vocal cords. Table 1 features the findings of her investigations conducted on the day of admission.

**Table 1:** Laboratory investigations of the patient AchR: Acetylcholine receptor

Investigation	Results	Normal range
Full blood count (FBC)		
White blood cells	6.08*10^3 ^/uL	4-10 *10^3^
Haemoglobin	12.7 g/dl	11-16
Platelets	290*10^3^/uL	150-140 *10^3^
Serum electrolytes (SE)		
Sodium	139 mmol/L	135-145
Potassium	4.1 mmol/L	3.5-5.5
Thyroid-stimulating hormone (TSH)	3.86 mIU/L	0.5-5
Erythrocyte sedimentation rate (ESR)	38mm/hr	Less than 30
Creatine phosphokinase (CPK)	39 U/l	26-192
Antibodies to AchR	Not detected	

The repetitive nerve stimulation test (RNST) showed a decremental pattern (Table 2).

**Table 2:** Results of the RNST RNST: Repetitive nerve stimulation test, ADM: Abductor digiti minimi, APB: Abductor pollicis brevis, L: Left, R: Right

Anatomy/train	Rate (Hz)	Amplitude (mV)	Amplitude (4%-1%)	F-wave amplitude (%)	Area (mVms)	Area (4%-1%)	F-wave area (%)
L ulnar: ADM							
Baseline	2	8.1	5.8	100	25.1	11.2	100
Post-10-second exercise	3	8.6	6.7	106	25.1	12.6	99.9
At 1 minute	2	8.5	4.4	105	24.3	9.1	96.9
Post-1-minute exercise	3	8.6	5.4	106	24.2	12.1	96.5
L median: APB							
Baseline	2	8.2	16.6	100	30.4	20.1	100
Post-10 second-exercise	2	6.8	-1.6	82.5	26.4	7.8	87
At 1 minute	3	8.8	25.7	108	33.4	27.8	110
L median: APB							
Baseline	2	7.4	32.7	100	27.4	30.9	100
Post 10-second-exercise	3	5.5	27.9	74.2	20.9	31.9	76.2
R median: APB							
Baseline	2	7.6	24.6	100	24.5	25.5	100
Post-10-second exercise	3	8.8	36.9	115	28.3	40.2	116
R accessory (spinal ): Trapezius							
Baseline	2	6.2	14.9	100	41.7	13.4	100
Post-10-second exercise	3	5.8	16.4	94.4	41.5	26.1	99.4
L accessory (spinal): Trapezius							
Baseline	2	6.9	15.5	100	44.0	20.1	100
Post-10-second exercise	3	6.9	7.2	100	41.9	20.4	95.1
At 1 minute	2	6.3	2.3	91.8	33.7	2.5	76.5
L ulnar: ADM							
Baseline	2	8.6	-2	100	20.0	-0.8	100
Post-10-second exercise	2	9.2	-2.6	106	20.4	2.4	102

There was no evidence of thymoma or thymic hyperplasia on contrast-enhanced computed tomography (CECT) of the chest (Video 1).

**Video 1:** The CECT of the patient's chest CECT: Contrast-enhanced computed tomography

Based on the clinical and investigation findings, the diagnosis of MG was made, and the patient was started on prednisolone and pyridostigmine. Mild clinical improvement of symptoms was noted with treatment; however, a satisfactory outcome was not achieved. There was clinical deterioration of her condition despite being on optimal doses of steroids and pyridostigmine. However, there was no respiratory compromise. She was started on intravenous immunoglobulin (IVIG) for five days. Her symptoms improved slightly with IVIG.

Due to predominant bulbar and facial muscle involvement, an unsatisfactory response to pyridostigmine, as well as prednisolone and AchR negativity, a MuSK antibody test was arranged, which resulted positive (6.82 nmol/L, normal <0.05). The patient was diagnosed with MuSK antibody-positive MG. She then received IV rituximab 500 mg weekly for four weeks. She noticed an improvement after the second dose of rituximab injection and made a remarkable recovery after one month of treatment.

## Discussion

Myasthenia gravis is an autoimmune disease characterized by autoantibodies that target components of the neuromuscular junction (NMJ), impairing neuromuscular transmission. About 80% of patients with MG have autoantibodies against AchR [6]. Less commonly identified antibodies include antibodies targeted to MuSK and low-density lipoprotein receptor-related protein 4 (LRP4).

When MG is associated with anti-MuSK antibodies, autoantibodies are produced against the MuSK protein, an IgG4 subtype, which plays a crucial role as a key regulator of synaptic differentiation at the NMJ. As a result of these antibodies, AChRs cluster poorly on the postsynaptic membrane, resulting in muscle weakness and defective neuromuscular transmission. Clinical presentation varies according to the type of autoantibody.

Predominant bulbar involvement has been demonstrated in about 80% of MuSK-MG patients, consisting of dysarthria, dysphonia with nasal speech, dysphagia, and weakness of muscles of mastication [1,5]. The bulbar onset of symptoms is usually related to rapid deterioration and early respiratory crises [5]. Axial muscle involvement is another common occurrence, particularly affecting neck extensors, which can be the only presenting symptom. Ocular manifestations other than ptosis, including ophthalmoplegia, can also be present. Typical fluctuation of symptoms may not be evident in MuSK-MG. Generalized weakness and fatigue resembling AchR MG have been described at the onset of the disease. When considering generalized muscle weakness, it's important to think about and exclude differential diagnoses such as motor neuron disease and myopathies.

Muscle-specific tyrosine kinase-positive MG can be diagnosed through clinical evaluation, serological testing, and electrophysiological tests. Predominant bulbar involvement, negative AchR antibodies, and AchR-negative patients who do not respond to conventional treatment should always alert the clinicians to the possibility of MuSK-MG, as in our patient.

Detection of MuSK antibodies is usually the second step in seronegative patients (AchR negative) and seropositive patients with poor response to treatment. The RNST and edrophonium/neostigmine tests have low sensitivity in diagnosing MuSK-MG. An RNST performed on proximal muscles, particularly fascial muscles, can yield a diagnostic sensitivity of 75% to 80% [1]. Single fiber electromyography (EMG) is the most sensitive diagnostic test for MuSK-MG. Since the beginning of the disease, single-fiber EMG of the cervical paraspinals, deltoid, frontalis, and orbicularis oculi, which are usually the first and most commonly involved muscles, may be noticeably abnormal. On the other hand, clinically uncompromised muscles may have normal jitter.

A multidisciplinary management approach is required to address MUSK-MG to control symptoms, modulate the immune system, and maintain functional status. Unsatisfactory responses to anticholinesterase medications have been reported by several studies [1,5]. A standard dose of pyridostigmine lacks efficacy in treatment and has poor tolerance due to cholinergic side effects. The mainstay of treatment is corticosteroid treatment, which is effective in almost half of the patients. In cases of life-threatening weakness or severe deterioration, high-dose prednisolone can be combined with plasma exchange or IVIG. Traditional immunosuppressants (azathioprine, mycophenolate mofetil (MMF)) are shown to be effective as steroid-sparing agents but exhibit lower rates of disease remission and a higher proportion of treatment dependence.

Evidence supports the use of monoclonal antibody therapy such as rituximab as an early therapeutic option in MuSK-MG patients with poor responses to steroids [1,7,8]. Patients with MuSK-MG receiving rituximab show sustained clinical improvement, as in our case. The dramatic response to rituximab could be attributed to MuSK antibodies being of the IgG4 subtype. There is no place for thymectomy in MuSK-MG. Evidence shows no benefit from thymectomy in the studied population [2,9].

## Conclusions

Muscle-specific tyrosine kinase antibody-positive myasthenia gravis presents as a unique and frequently more severe subtype of MG, characterized by an acute onset of bulbar symptoms with rapid progression within weeks. Timely diagnosis and utilizing MuSK-antibody testing are essential for early intervention. Corticosteroids, plasmapheresis, and rituximab remain key components of management despite the challenges in treatment response.
